# Population Structure and Genetic Diversity of the *Fusarium graminearum* Species Complex

**DOI:** 10.3390/toxins3081020

**Published:** 2011-08-19

**Authors:** Jian-Hua Wang, Mbacke Ndoye, Jing-Bo Zhang, He-Ping Li, Yu-Cai Liao

**Affiliations:** 1 Molecular Biotechnology Laboratory of Triticeae Crops, Huazhong Agricultural University, Wuhan 430070, Hubei, China; Email: jianhuawang@webmail.hzau.edu.cn (J.-H.W.); n.mbacke@gmail.com (M.N.); jingbozhang@mail.hzau.edu.cn (J.-B.Z.); hepingli@mail.hzau.edu.cn (H.-P.L.); 2 College of Plant Science and Technology, Huazhong Agricultural University, Wuhan 430070, Hubei, China

**Keywords:** *Fusarium graminearum *species complex, chemotype, genetic diversity, population structure, geographic distribution

## Abstract

The *Fusarium graminearum* species complex (*Fg* complex) consists of phylogenetically distinct species some of which cannot be discriminated based on their morphology. Their chemotypes and geographic distributions are dramatically different, and these highlight the challenges that *Fusarium* head blight (FHB) poses to plant disease specialists and plant breeders, thereby requiring that quarantine officials employ molecular diagnostic tools in their active surveillance programs. Molecular marker technologies play essential roles in species identification of the *Fg* complex, and they are being used widely to assess the genetic diversity of the clade. The utility, applicability and limitations of molecular methods for assessing the population structure and genetic diversity within the *Fg* complex are discussed with suitable examples. Knowledge gained from these studies will provide a baseline for monitoring changes in FHB pathogen diversity and mycotoxin potential over time, both of which are critical to the ultimate control and elimination of this economically devastating disease.

## 1. Introduction

Filamentous fungi within the *Fusarium graminearum* species complex (*Fg* complex) are the most important etiological agents of *Fusarium *head blight (FHB) on wheat and other cereal grains worldwide [1,2,3]. Over the past decade, the *Fg* complex has emerged as one of the most destructive pathogens of cereals resulting in losses valued at billions of dollars to the grain industry [4,5]. Besides reducing yield, these fungi can cause further losses through contamination of crops with mycotoxins such as deoxynivalenol, nivalenol and zearalenone [6,7], which pose a serious threat to animal health and food safety.

Members of the *Fg* complex appear to have evolved allopatrically [8,9,10,11]. Biogeographic data suggests that the majority of species within the *Fg* complex have evolutionary origins in the Southern Hemisphere [2,3] and Asia [9]. During recent years, changed agronomy, trade and shipment of cereal commodities, and global climate change have aggravated the spread and severity of FHB northward in China [12,13,14] and also have resulted in the reemergence of FHB to epidemic levels in the United States, Canada and Europe, causing losses of about $3 billion for the United States alone between 1998 and 2000 [15,16]. It seems likely that some *Fg* complex species have been introduced into non-indigenous areas relatively recently, especially in areas that rely extensively on agricultural imports [2,3,17,18,19,20,21,22,23,24,25]. 

In view of the importance of diseases caused by members of the *Fg* complex, procedures for effective management of these pathogens are urgently needed. In this context, information on the population genetic diversity and dispersal of the *Fg* complex is, consequently, of importance. Various genotypic and phenotypic approaches have been in use over the past few years in research pertaining to the population genetic structure of the *Fg* complex [1,26,27,28,29,30,31,32,33].

This review seeks to outline some of the findings of genetic diversity studies on the *Fg* complex that have been conducted over the past two decades. It is not meant to be a comprehensive review of the literature but rather a reflection on some of the common approaches, assumptions, and findings in the field. The genetic diversity, population structure and geographic distribution of the *Fg* complex that have been the focus of these studies will be reviewed. In addition, potential topics for future research will be discussed.

## 2. Population Structure and Geographic Distribution of the *Fg* Complex

Natural occurrence of the *Fg* complex causing FHB on cereal crops have been reported in different continents, e.g., Africa [11,34,35,36,37], America [3,4,7,17,20,25,33,38,39,40,41,42,43,44,45,46,47,48,49], Asia [9,13,14,17,21,22,23,24,28,30,50,51,52,53,54,55,56,57], Europe [50,58,59,60], and Oceania [18,61,62,63,64]. Members of the *Fg* complex were considered a single cosmopolitan species, due to the failure of morphological species recognition to accurately assess species limits for this group. O’Donnell *et al*. [1] first identified seven phylogenetic lineages within the *Fg* complex, employing genealogical concordance phylogenetic species recognition (GCPSR) [65]. Most recently, employing a high-throughput multilocus genotyping (MLGT) assay of portions of 13 housing keeping genes, combined with GCPSR and molecular marker technologies, 13 phylogenetically distinct, cryptic species have been identified within the *Fg* complex [1,2,3,9,11]. The species designation *Fusarium graminearum* will therefore be *sensu stricto*, and other *Fg *complex species designations will be used. These 13 species include: *F. austroamericanum*, *F. meridionale*, *F. boothii*, *F. mesoamericanum*, *F. acaciae-mearnsii*, *F. asiaticum*, *F. graminearum sensu stricto*, *F. cortaderiae*, *F. brasilicum*, *F. gerlachii*, *F. vorosii*, *F. aethiopicum*, and *F. ussurianum* [1,2,3,9,11]. In addition, a putative new species within the *Fg* complex was identified in Nepal by Chandler *et al.* [10] and later characterized by Desjardins and Proctor [66] as the “Nepal lineage”.

A biogeographic hypothesis suggests that the basal most species within the *Fg* complex may be endemic to the southern hemisphere while the derived species evolved within the northern hemisphere [3]. According to the surveys to date, *F. austroamericanum*, *F*. *meridionale*, *F*. *cortaderiae*, and *F*. *brasilicum* appear to be endemic to South America [3,46]; *F*. *acaciae-mearnsii *to Australia or less likely Africa [2]; *F*. *asiaticum*, *F*. *vorosii* and *F*. *ussurianum* to Asia [2]; *F*. *aethiopicum* to Africa [11]; *F. boothii* and *F*. *mesoamericanum* to Central America [2,11]; *F*. *gerlachii *to the US [3].

In the surveys conducted worldwide to date, *F. graminearum sensu stricto* (*F. graminearum s.s.*) is cosmopolitan in distribution and has been found in Asia, Africa, America, Europe, and Oceania, while another species, *F. asiaticum*, is widespread in Asia [1,2,59,41,66,54]. FHB was initially reported in 1936 in China, and since then FHB epidemics have become more severe and frequent in the middle and lower regions of the Yangtze River, and in Heilongjiang province in northeastern China [12]. More recently FHB frequently occurs in areas along the Yangtze River, a region with conditions favorable for infection by the pathogen [13,14,21,22,23,24]. *F. asiaticum* is the predominant species in this region [13,14,30,21,22,23,24]. Based on the climate data of 30 y during 1970–1999, our group assayed the geographical distribution patterns of *F. graminearum s.s.* and *F. asiaticum *in China. Our results indicated that *F. graminearum s.s.* was mainly obtained from cooler regions where the annual average temperature was 15 °C or lower. In contrast, 95% of *F. asiaticum* isolates were collected from warmer regions [14,21]. These results suggest ecological factors may have had a significant effect on the distribution of these species [13,14,46,51,54,66]. In Japan, *F. graminearum s.s.* and *F. asiaticum* are the predominant species, with *F. asiaticum* predominating in the southern regions as in China [52]. A preliminary report of the *F. graminearum* clade isolated from maize in Korea indicated that *F. graminearum s.s.* was dominant, accounting for 74% of strains, while three additional species, *F. meridionale*, *F. boothii*, and *F. asiaticum*, were also present at low frequencies [67]. However, *F. asiaticum*, *F. meridionale* and *F. boothii* were the major causal agents of *Gibberella* ear rot of maize in Nepal, whereas *F. graminearum s.s.*, which dominates in maize elsewhere in Asia and worldwide, was not detected [66]. Similarly, *F. meridionale* and *F. boothii* play a substantial role in the infection and trichothecene contamination of maize in Argentina. *F. graminearum s.s.* was not represented among the isolates examined [46]. In contrast, a previous survey of the *Fg* complex collected from wheat in the center of Argentina identified all 113 isolates as *F. graminearum s.s. *[41]. In Korea, 80% of the isolates collected from rice belonged to *F. asiaticum*, which is dramatically different from the results on maize [54]. Although the underlying factors for species distribution are unknown, the occurrence of *F. asiaticum *in Louisiana closely overlaps with rice-growing areas in Louisiana [25]. 

## 3. Chemotype Distribution of the *Fg* Complex

Every species within the *Fg* complex is capable of producing type B trichothecenes in planta [11]. Based on the chemical structure, including the acetylation position, three type B trichothecene chemotypes have been identified within the *Fg* complex: deoxynivalenol and 3-acetyldeoxy-nivalenol (3-AcDON), deoxynivalenol and 15-acetyldeoxynivalenol (15-AcDON), and nivalenol and 4-acetylnivalenol (NIV) [32,68,69]. 

In the limited surveys to date, strains of several newly discovered species were found to represent only a single chemotype: 3-AcDON for the *F*. *ussurianum* strains [9], 15-AcDON for *F*. *aethiopicum*, *F. boothii* and *F*. *vorosii* strains [3,9,11,20,70], and NIV for *F. meridionale* and *F*. *gerlachii* strains [3,11,20,46,70]. 3-AcDON, 15-AcDON and NIV producers were identified in both *F. graminearum s.s.* and *F. asiaticum*. The remaining 5 species, *F. acaciae-mearnsii*, *F*. *mesoamericanum*, *F*. *austroamericanum*, *F*. *cortaderiae*, and *F*. *brasilicum* can produce both 3-AcDON and NIV [20].

Dramatic differences in the geographic distribution of *Fg* complex chemotypes were reported by Gale *et al.* [30], Zhang *et al.* [21] and Qu *et al.* [13] in China, and by Karugia *et al.* [56] in Japan. Results of these surveys show that Chinese populations of *F. graminearum s.s.* on wheat and barley produce 15-AcDON predominately or exclusively, in contrast to populations of *F. asiaticum*, which predominately produce 3-AcDON or NIV. While in Japan, out of a total of 179 *F. asiaticum* isolates, 80 isolates were of the NIV chemotype, and the remaining 99 isolates were of 3-AcDON producers [56]. NIV chemotype was predominant among the *Fg* complex strains isolated from Korean rice [54,57]. In contrast, the predominant chemotype among the corn isolates varied according to geographic region [57]. In Nepal, NIV producers of the *Fg* complex were isolated more than twice as frequently as DON producers from maize seed samples [66,71].

Direct competitive enzyme-linked immunosorbent assay (ELISA) indicated that 75% of grain samples from Kenya were contaminated with DON [35]. Eleven *Fusarium*-related mycotoxins were quantified by chemical analysis, the results demonstrated that DON was detected at the highest frequency (69%) [36]. The trichothecene chemotype of 560 South African isolates of the *Fg* complex collected from diseased wheat, barley and maize were determined using MLGT assay. The results showed that 535 isolates possessed the 15-AcDON chemotype, 22 isolates represented the NIV chemotype, and only 3 isolates were of the 3-AcDON chemotype [37]. *F. aethiopicum*, a novel species reported only from Ethiopia, belonged to 15-AcDON chemotype [11,37].

In the surveys conducted to date in Europe, the 15-AcDON chemotype of the *Fg* complex was the predominant, followed by the 3-AcDON chemotype and a few isolates of the NIV chemotype. For example, in UK [72], Germany [73], and Luxembourg [74], the 15-AcDON chemotype represented more than 90% of the population, and DON producers also were in high portion in Netherlands [58]. In New Zealand, however, Lauren *et al.* [62] and Monds *et al.* [18] found that among the *F. graminearum s.s.* strains, NIV chemotypes were as common as DON chemotypes, with the DON chemotypes producing primarily 15-AcDON. All the isolates of *F. cortaderiae*, the other *Fg* clade species found in New Zealand, were NIV producers [18].

Previous studies indicated that *F. graminearum s.s.* isolates with a 15-AcDON chemotype represented the predominant cause of FHB in North America [68,75,76]. However, a significant increasing frequency of 3-AcDON chemotype from west to east occurred in Canada in the last decade [20,77]. Schmale *et al.* [78] evaluated the trichothecene genotypes of 998 *Gibberella zeae *(*G. zeae*) isolates gathered from 39 winter wheat fields in the eastern US, and found that 92% percent of the isolates were 15-AcDON, 7% were 3-AcDON, and 1% was NIV, with an increasing trend of 3-AcDON producers from south to north. Starkey *et al.* [3] identified 2 NIV-chemotype isolates (NRRL 38371 and NRRL 38383) within 6 *F. graminearum s.s.* strains in Louisiana. A recent survey by Gale *et al.* [25] suggests that NIV-type *F. graminearum s.s.* populations represented a high proportion (79%) of isolates in Louisiana. It was suggested that the NIV chemotype of *F. graminearum s.s. *was rare in the United States, and that the only way for the NIV chemotype with adequate fitness to become established in North America would be by migration and introgression into the resident DON populations [71]. In addition, chemotype variation has been maintained by natural selection and appears to have adaptive potential for FHB pathogens [32]. 

In Argentina, within the *Fg* complex strains, 15-AcDON chemotype was the most common [44,47], and trichothecene chemotype diversity among the isolates examined was directly tied to species differences [46]. Two studies of the wheat kernel samples in southern Brazil showed that 15-AcDON chemotype was predominant (>92%), followed by the NIV chemotype, and no 3-AcDON chemotype was observed [43,49]. A recent survey of barley grain samples revealed that, 4% of the strains were 3-AcDON chemotype, 66% were 15-AcDON chemotype and 29% were NIV chemotype [48].

Broad geographic surveys for trichothecene chemotype of the *Fg* complex from cereal crops are important to establish baselines for these genotypes against which any future shifts in populations can be assessed [3,20,25,42,77,78].

## 4. Molecular Markers for *Fg* Complex Genetic Analyses

High genotypic diversity has been revealed within the Fg complex, even within a species [30,33,38,56,79,80], using molecular markers. Molecular markers such as sequence characterized amplified regions (SCAR), single strand conformational polymorphism (SSCP), randomly amplified polymorphic DNA (RAPD), amplified fragment length polymorphism (AFLP), restriction fragment length polymorphism (RFLP), sequence related amplified polymorphism (SRAP), single nucleotide polymorphism (SNP), and variable number of tandem repeat (VNTR) have been used extensively to study genetic variability of the Fg complex. Recently, a high resolution, SNP-based multilocus genotyping assay was used in numerous investigations of diversity within the Fg complex, and several novel species have been identified.

### 4.1. SCAR (Sequence Characterized Amplified Regions) or SSCP (Single Strand Conformational Polymorphism)

SCAR analysis using Fg16F/R primer was previously used to distinguish *F. graminearum  s.s.* and *F. asiaticum* isolates from China [14]. However, differentiation of *F. asiaticum* from *F. meridionale* on the basis of SCAR fragment size was not reliable because the Fg16F/R amplicon size is identical (497 bp) [29]. However, because the sequence of the *F. meridionale* and *F. asiaticum* amplicons differ, single-strand conformational polymorphism (SSCP) enabled unequivocal resolution of *F. meridionale* from *F. asiaticum* [13]. 

SSCP analysis of the Fg16F/R PCR amplicon differentiated *F. graminearum  s.s.*, *F. asiaticum *and *F. meridionale *from China and revealed three haplotypes among sequence-characterized amplified region (SCAR) type 1 *F. graminearum s.s.* isolates: 1A, 1B and 1C, while the remaining SCAR types displayed the same SSCP patterns with no polymorphism [13]. All the *F. graminearum  s.s.* isolates from China and Europe, and 5 of the seven isolates from the US were haplotype 1A. The remaining two American isolates (A2 and A5) were haplotype 1B [13]. DNA sequencing of the Fg16F/R product confirmed that haplotype 1B differed from that of 1A at nucleotide 348 (C-T) and 412 (T-A) [81]. SSCP assays of a further 440 isolates from China indicated that 90 were *F. graminearum *(haplotype 1A) and 350 were *F. asiaticum *(haplotype 5) with no polymorphism within the species [81]. This high level of uniformity contrasts markedly with isolates from elsewhere. For example, three haplotypes (haplotypes 3, 4 and 5) were observed among *F. asiaticum* isolates from Nepal [29], while two haplotypes were observed among the limited number of isolates from Europe (haplotypes 1A and 6) and the US (haplotypes 1A and 1B). 

Investigators have used a variety of methods to improve the resolving power of SSCP, however, differentiation among polymorphic molecules on a polyacrylamide matrix is not entirely predictable, and the method can result in false negatives, ambiguous results and experimental artifacts. The low haplotype diversity detected by the SSCP markers indicates that it is too conserved for population genetic studies of the *Fg* complex [14].

### 4.2. RAPD (Randomly Amplified Polymorphic DNA)

RAPD technique is a PCR procedure involving very short (10 or fewer bases) arbitrary primers at a low annealing temperature (30–38 °C). RAPD analysis detects two types of genetic variation: (i) in the length of DNA between the two primer binding sites; and (ii) in sequence variation at the priming regions. When the amplified products from such a reaction are analyzed on an electrophoresis gel, unique banding patterns are seen. These patterns may reflect the differences characteristic of certain species or strains. The RAPD method is simple, sensitive, rapid and has been used for distinguishing several pathogens [82]. A high level of genotypic diversity in *Fg* complex isolates in Canada, via RAPD markers, was demonstrated by Dusabenyagasani *et al*. [83], and 90.56% of the genetic variability was explained by within-region variation. RAPD analysis of 42 isolates, which originated from northwest Europe, the US and Nepal, identified three groups, two of which contained the isolates from Nepal, and a third contained the isolates from Europe and the US [29]. A considerable genetic resemblance was found by RAPD between 34 isolates from northeastern and northwestern China. The isolate grouping was not related to pathogenicity or to host cultivar [84]. Similar findings were obtained in the US, where *F. graminearum s.s.* is the predominant FHB species [3,38,42]. However, relatively low amount of genetic diversity were revealed using RAPD analysis of isolates in Canada [85] and Brazil [86].

RAPD has the major disadvantage that it is very sensitive to the reaction conditions, DNA quality and PCR temperature profiles [87], which limits its application. This problem can be minimized if strains under study are treated identically. RAPD has been criticized for lack of reproducibility [88].

### 4.3. AFLP (Amplified Fragment Length Polymorphism)

AFLP is a DNA fingerprinting technique that detects genomic restriction fragments and thus resembles the RFLP technique. The method allows for the specific co-amplification of high numbers of restriction fragments. Typically 50–100 restriction fragments are amplified and detected on denaturing polyacrylamide gels. 

The advantage of AFLP over other techniques is that multiple bands are derived from all over the genome. This prevents overinterpretation or misinterpretation due to point mutations or single-locus recombination, which may affect other genotypic characteristics. Different enzymes and/or selective extension nucleotides can be used to create new sets of markers. Therefore, AFLP can provide an almost limitless set of genetic markers, and as many as 50–75 AFLP markers can be resolved per reaction [89]. The AFLP markers showed a higher degree of resolution for discriminating closely related fungi. In *Fusarium* taxonomy, AFLP was first applied to discriminate between different strains of the *Fg* complex [90]. It was used for assessing the population structure of the *Fg* complex and identified considerable diversity within the clade [14,91,92,93]. Bowden *et al.* [94] using AFLP analysis to determine genetic diversity in the *Fg* complex from Kansas and North Dakota, found a high degree of homogeneity between subpopulations. The analysis of 59 isolates from Australia by AFLP showed that 56 isolates had distinct haplotypes, and the spatial diversity within the *F. graminearum s.s.* strains was high within a single field [63]. The AFLP analysis by Monds *et al.* [18] showed that the *Fg* complex was represented by *F. graminearum* *s.s.* and *F. cortaderiae* in New Zealand, and that these two species originated from at least two regions, and probably on at least two hosts. More diversity of the *Fg* complex was detected by AFLP analysis of populations from Europe, the US or Nepal [14]. Based on AFLP analyses, 270 bands were detected among 333 *F. asiaticum* isolates from the five populations in Korea, and 36% of the AFLP bands were polymorphic [54]. Among the 270 multilocus haplotypes, 225 were represented by a single strain, and 45 haplotypes were detected more than once. Genotypic diversity varied by population, and the number of polymorphic loci in an individual population ranged from 32 to 56 [54]. A sample of 103 *F. graminearum* *s.s.* isolates from Brazil were assessed employing AFLP. Astolfi *et al.* [49] found that isolates with the same haplotype were rare and genotypic diversity was uniformly high (≥98% of the count) in all three subpopulations analyzed. 

In our experience, AFLP is not highly reproducible. When controlled experiments are conducted over 10% of the bands are not reproduced. Also scoring band size is exceedingly difficult. Besides, alleles are not easily recognized [95], even when a capillary DNA sequencer is employed [96].

### 4.4. SRAP (Sequence Related Amplified Polymorphism)

Sequence-related amplified polymorphism (SRAP) is a novel molecular marker technique based on two-primer amplification that preferentially amplifies open reading frames (ORFs) [97]. The forward primers preferentially amplify exonic regions, and the reverse primers preferentially amplify intronic regions and regions with promoters. The observed polymorphism originates in the variation in the length of these extrons, introns, promoters, and spacers, both among individuals and among species [97]. With this unique primer design, SRAP markers are more reproducible, more stable, and less complex than RAPD and AFLPs. This technique has been used to investigate genetic diversity of the *Fg* complex in Canada [40]. SRAP results are in agreement with findings of high diversity of the *Fg* complex isolates from the United States and eastern Canada based on RAPD and AFLP markers [98]. It could help to assess whether different management practices affect the genetic diversity of the *Fg* complex populations [98].

The disadvantage of this technique is that both dominant and co-dominant markers can be produced which makes the interpretation of the data a little bit more complicated.

### 4.5. SNP (Single Nucleotide Polymorphism)

A SNP is a single base pair mutation at a specific locus, usually consisting of two alleles that can be amplified by two pairs of primers in one PCR reaction. Cuomo *et al*. [99] identified more than 10,000 SNPs from a comparison of two *F. graminearum s.s.* strains (strains PH-1 and GZ3639), which were frequently located near telomeres and within other discrete chromosomal segments. *F. graminearum* genes specifically expressed during plant infection (including predicted secreted proteins, major facilitator transporters, amino acid transporters, and cytochrome P450s) are all overrepresented in high SNP density regions, which may allow the fungus to adapt rapidly to changing environments or hosts. Perhaps the SNP have the greatest potential in elucidating the dynamics of host pathogen interactions. 

Yang *et al*. [22] characterized *Fusarium* isolates at the species level by a robust set of diagnostic primers based on SNPs among members of the *Fg* complex. In addition, numerous SNPs-based trichothecene chemotype assays were developed from the trichothecene biosynthetic pathway genes. Two sets of multiplex primers specific to individual chemotypes were designed from the *Tri3* and *Tri12* genes [20,32]. Both multiplex assays were widely used to predict the chemotype of the *Fg* complex strains [3,25,43,48,49,78,100]. Primers derived from the *Tri13* and *Tri7* genes [10,58], which are responsible for the oxygenation and acetylating of the C-4 atom of the trichothecene molecule [101], were used to discriminate between DON and NIV producers [10,43,51,53,58,72,100]. The primer pairs Tri303F/R and Tri315F/R derived from the *Tri3* gene is used to predict if a DON chemotype is a 3-AcDON or 15-AcDON producer [21,51,72]. Very recently, Zhang *et al.* [23] also developed an effective set of diagnostic primers based on SNPs among three different chemotype isolates and determined that there was a recent shift to the 3-AcDON chemotype. The high genetic diversity of this group of genes suggests that the fungus has great capacity for adaptability and genetic change during its interaction with a host species. The richness in SNPs in the fungal genome combined with real-time PCR assays would provide a powerful tool for development of markers for various identifications within the *Fg* complex.

### 4.6. VNTR (Variable Number of Tandem Repeat)

VNTR markers have been developed for *Fg* complex species and have been effectively used for population analyses [20,55,70,102]. VNTR may be more desirable than RFLP markers for population genetic analysis due to their ability to generate accurate polymorphic data and due to their codominance. Generally, the development of VNTR markers is costly and burdensome and therefore few VNTR markers have been reported to date for the *Fg *complex [102,103]. A relative small genome size, 36.1 Mb, of *F. graminearum s.s.* (strain PH-1, NRRL 31084) sequenced recently by the Center for Genome Research, Cambridge, MA, USA [99], may carry a low level of variation within tandem repeat sequences in the genome. However, the availability of the whole genome sequence may allow a bioinformatic approach for the further development of VNTR markers in these species. Zhang *et al*. [23], using seven highly informative VNTR markers on 448 *F. asiaticum* isolates from Chinese barley, showed a significant degree of population subdivision (*P* < 0.001) among populations from the upper, middle, and lower valleys of the Yangtze River, with little gene flow (*Nm* = 1.210). A comprehensive VNTR analysis of 1106 *F. asiaticum* isolates collected from western China, centre and eastern provinces, indicated that the genetic diversity of the species showed a clear substructure even within provinces [24]. Ten VNTR markers were used to determine the genetic diversity of an older population (*n* = 115 isolates) of *G. zeae* collected from 1997 to 2000 with a newer population (*n* = 147 isolates) collected in 2008. High gene diversity and genotypic diversity were revealed, but the linkage disequilibrium were low in both populations, and the differentiation between the two populations was low [100]. 

### 4.7. RFLP (Restriction Fragment Length Polymorphism)

The RFLP method is used to assess the extent of natural variation or relatedness in the genomes of different species, biotypes, strains or races of fungal pathogens. Deletions or insertions in the DNA sequences may lead to variation (polymorphisms) in fragment sizes. The sensitivity and reliability of the assay may be enhanced by employing highly repetitive DNA sequences as probes, as the signal is present in multiple copies.

Members of the *Fg* complex and *F. pseudograminearum* are morphologically very similar, and were previously designated Group 1 and Group 2 of *F. graminearum* [104]. RFLP [105], DNA sequence [106], and isozyme [107] data indicated that these two groups were phylogenetically distinct and should be regarded as separate species. RFLP analysis of the *Fg* complex collected from eastern China showed a population mean gene diversity of 30.6% to 36.4% and identified 65 distinct haplotypes among 225 isolates [30]. 

An alternative PCR-RFLP marker system based on single-copy nuclear polymorphic (SCNP) sequences in *Fg* complex was developed [25]. These markers can be useful when VNTR analysis is technologically not possible, mutation rates differ, or selective neutrality is a concern [25]. This method not only substantially reduced screening time and cost but also allowed for the strategic positioning of markers across the whole genome. Based on the moderate number of alleles per locus and the low number of shared genotypes, this PCR-RFLP system allows for excellent genotypic resolution without excessive polymorphisms [25]. Therefore it is suitable for population level analysis of the *Fg* complex in the future.

### 4.8. GCPSR (Genealogical Concordance Phylogenetic Species Recognition) and MLGT (Multilocus Genotyping Assay)

GCPSR was first used to investigate species diversity within the Fg complex by O’Donnell et al. [1]. A global collection of FHB strains were investigated by Ward et al. [32], O’Donnell et al. [2], and Starkey et al. [3], phylogenetic analyses of DNA sequences from portions of 13 independent loci, including translation elongation factor (EF-1α), α-tubulin (α-tub), β-tubulin (β-tub), histone H3 (HIS), phosphate permease (PHO), reductase (RED), trichothecene 3-O-acetyltransferase (Tri101), ammonium ligase (URA), ITS/28S rDNA locus, and four adjacent mating-type (MAT) genes (MAT1-1-3, MAT1-1-2, MAT1-1-1, and MAT1-2-1) plus the corresponding intergenic regions (A, B, and C), totaling 16.33 kb, provided strong evidence that this morphologically defined species comprises at least 13 phylogenetically distinct species. 

MLGT was developed by Ward et al. [20]. Forty-one oligonucleotide probes targeting species or trichothecene chemotype-specific variation within 6 genes were developed base on SNPs [20], which facilitated the species and chemotype assays of the Fg complex. Based on the MLGT assays, two novel species within the Fg complex, F. aethiopicum [11] and F. ussurianum [9], were recently identified in Ethiopia and the Russian Far East, respectively. Four novel probes, 2 for F. aethiopicum and 2 for F. ussurianum, were developed from variation identified in the Tri101 and RED genes for identification of these two species [9,11]. MLGT is based on direct interrogation of SNPs. It provides a rapid, efficient and accurate framework for studies to understand the population dynamics, trichothecene chemotype distribution, and ecology of the Fg complex [9,11,20,25,37,45].

## 5. Conclusions

Members of the *Fg* complex were thought to represent the single species *F. graminearum* (teleomorph *G. zeae*) [11]. Thus multiple *Fg* complex were reported under the name *F. graminearum* in previous studies. Morphological variation within the *Fg* complex is insufﬁcient for discerning species identity. However, by employing GCPSR, a MLGT assay, and different molecular marker technologies, 13 phylogenetically distinct species have been identified within the *Fg* complex [1,2,3,9,11,65]. These findings indicate that diversity within the *Fg* species complex is much greater than previously believed [46].

As FHB is a serious threat to cereal production worldwide, information on the global distribution, aggressiveness, and toxin accumulation of *Fg* complex strains will be critical to identifying and implementing pathogen control strategies. Some updated molecular marker technologies, such as VNTR and PCR-RFLP, will play an important role in population genetic analyses of the *Fg* complex. The increasing database of global surveillance information is providing a detailed understanding of the geographic distribution, host range, aggressiveness, epidemiology, and evolutionary dynamics of the FHB pathogens and their toxin potential, all of which are critical for monitoring changes in this economically devastating disease worldwide [11]. 
